# The Effect of Crumb Rubber Particle Size to the Optimum Binder Content for Open Graded Friction Course

**DOI:** 10.1155/2014/240786

**Published:** 2014-01-16

**Authors:** Mohd Rasdan Ibrahim, Herda Yati Katman, Mohamed Rehan Karim, Suhana Koting, Nuha S. Mashaan

**Affiliations:** ^1^Centre for Transportation Research, Faculty of Engineering, University of Malaya, 50603 Kuala Lumpur, Malaysia; ^2^Universiti Tenaga Nasional, Putrajaya Campus, Jalan Ikram-Uniten, 43000 Kajang, Selangor, Malaysia

## Abstract

The main objective of this paper is to investigate the relations of rubber size, rubber content, and binder content in determination of optimum binder content for open graded friction course (OGFC). Mix gradation type B as specified in Specification for Porous Asphalt produced by the Road Engineering Association of Malaysia (REAM) was used in this study. Marshall specimens were prepared with four different sizes of rubber, namely, 20 mesh size [0.841 mm], 40 mesh [0.42 mm], 80 mesh [0.177 mm], and 100 mesh [0.149 mm] with different concentrations of rubberised bitumen (4%, 8%, and 12%) and different percentages of binder content (4%–7%). The appropriate optimum binder content is then selected according to the results of the air voids, binder draindown, and abrasion loss test. Test results found that crumb rubber particle size can affect the optimum binder content for OGFC.

## 1. Introduction

The use of OGFC which is also known as porous asphalt started in the 1930s when Oregon's Department of Transportation applied the open graded design in an attempt to improve the skid resistance of its road. However, at that time, durability and draindown problems curtailed its further usage. In the 1940s, California Department of Transportation (CALTRANS) utilized this type of surface course as drainage interlayer and as an alternative to chip seals and slurry seals [1]. The first guide in designing porous asphalt mixes was published in 1974 by the Federal Highway Administration agency [2] and, in 1978, The Franklin Institute in Philadelphia published a guideline in designing porous pavements which includes considerations in soil conditions, load bearing capability, and hydrological concerns of the design [3]. The popularity of this type of pavement is due to a number of advantages it has such as reduction in splash and spray, reduction of the risk of wet skidding, and hydroplaning with lower noise generated from the traffic friction. These benefits make it gain popularity with time and more authorities had started using the system until the present day. In tropical countries such as Malaysia, where the average monthly precipitation can be as high as 314 mm, [4] the use of open graded pavement system can be extremely beneficial. However, the advantages also come with a list of disadvantages such as reduced structural and functional durability and higher construction and maintenance costs. This calls for continuous research [5, 6] in this field in order to improve the shortcomings of the design.

Open graded friction course or porous asphalt system consists of a layer of porous asphalt, a layer of granular subbase, and a layer of crushed stone base course that acts as a reservoir [3]. This allows water to infiltrate the surface course and be stored at the base before the water reaches the subgrade. Due to its high porosity, porous asphalt can face problems such as faster oxidation rate of binder and loss of adhesion due to contact with water. This accelerates the disintegration process which contributes to raveling problem [2, 7]. This calls for modified binder to be used, as modified binder can improve the durability characteristics of the pavement. Currently, the majority of authorities that uses open graded asphalt in their roads opted for either rubber or polymer modification to increase the durability of the pavement [5].

Crumb rubber modification by the wet process and dry process [8] has been shown to have the ability to improve the rutting resistance, resilience modulus, and fatigue cracking resistance of asphaltic mixes. This is due to the alteration of the property of the bituminous binder in terms of the viscosity [9], softening point [10], loss modulus, and storage modulus [11]. The improvement, however, is governed by the swelling process of rubber particles that were interacted with bitumen. Rubber crumbs can swell up to 3 to 5 times its original size due to the absorption of maltenes component of the bitumen [12, 13]. This left a higher proportion of asphaltenes in the binder, therefore increasing its viscosity.

The outcome properties of crumb rubber modified bitumen (CRMB) are very sensitive and are highly governed by the mixing process which is dependent on external factors such as the mixing temperature, mixing duration, and type, and internal factors such as type of bitumen, crumb rubber quantity, particle size, and type [14]. Accurate selection of the processing variables such as bitumen type, rubber particle size and content, mixing temperature, and duration is the key to successful CRMB production.

This paper attempts to correlate the relationship between crumb rubber content and particle size with the optimum binder content of an open graded friction course manufactured according to the Specifications for Porous Asphalt produced by the Road Engineering Association of Malaysia (REAM) modified with crumb rubber by the wet process.

## 2. Optimum Binder Content for Porous Asphalt

A number of guides for designing open graded friction course had been published worldwide by research institutes and road and transport authorities alike. There are generally three main methods of determining optimum binder content for porous mixes. The first type determines the optimum binder content using compacted asphalt specimen, while the second uses an oil absorption test and the third type uses visual observation [2]. This paper focuses on the first method where the optimum binder content of OGFC is determined using physical characteristics compacted specimens.

Generally, the guides that use compacted specimens to determine optimum binder contents require the design to balance between the abrasion loss of the mix and binder draindown values while retaining adequate amount of air void in the mix to provide good permeability. The bitumen content that produces samples that fit all the required criteria is taken as the optimum bitumen content. Apart from the above main parameters, some guides require mix samples to be tested for aged abrasion loss as well.  Table 1 summarizes the main governing parameters that were specified in some of the design guides to determine optimum asphalt content.

## 3. Materials

All experiments and materials were conducted in compliance with the Road Engineering Association of Malaysia and Malaysian Public Works Department (REAM-SP 5/2008), the American Society of Testing and Materials (ASTM), and the British Standard (BS). To ensure the same characteristics of materials, the source of supply and specifications of the material were maintained.

### 3.1. Aggregates

**Figure 1:**
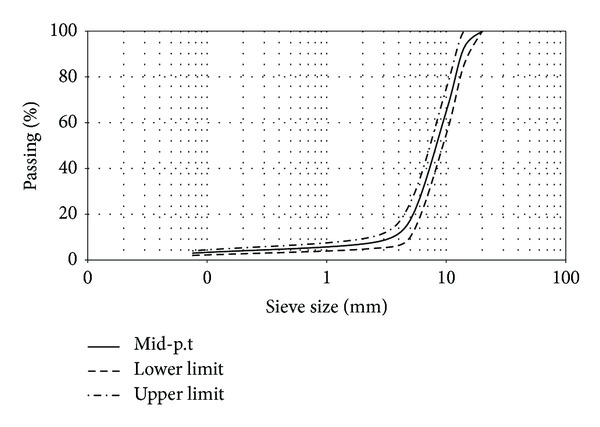
Aggregate grading for porous asphalt specimen.

Aggregate used was a mixture of coarse aggregate, fine aggregate, and mineral filler. In this study, Portland cement was used as mineral filler. Porous asphalt mix gradation type B adopted from REAM-SP 5/2008 as shown in Figure 1 and Table 2 was used in all samples preparation.

### 3.2. Bitumen

The binder used was 80/100 penetration grade bitumen which had consistency of 89 penetrations at 25°C and ring and ball softening point of 48°C. Both were tested according to ASTM D36-95 (ASTM, 1999) and BSI 2000: Part 58: 1988 (BSI, 1988), respectively.

### 3.3. Rubberized Bitumen

Different sizes of rubber crumb, namely, 20 mesh [0.841 mm], 40 mesh [0.42 mm], 80 mesh [0.177 mm], and 100 mesh [0.149 mm] were used in the preparation of the rubberised bitumen that were later used to prepare the Marshal samples. In the preparation of rubberized bitumen, four different additive concentrations were used to see the effect of rubber contents on the performance of the mixes. The amounts of rubber crumb used were 4%, 8%, and 12% by weight of the bitumen. The mixing process was done using a propeller mixer at 200 rpm with the temperature of 160°C for one hour.

## 4. Experimental

### 4.1. Determination of Optimum Binder Content

Samples preparation was followed closely to ASTM D6926: Practice for Preparation of Bituminous Specimens Using Marshall Apparatus. To determine the optimum binder content, three parameters need to be determined, namely, the air void in the compacted mix (D2041 and D 3203), the Binder Drainage Test (BS EN 1297-18:2004), and the Cantabro Abrasion Test (D7064) of the mix. The Malaysian Standard for Porous Asphalt (REAM SP 5/2008) requires mixes to have void content that is between 18% and 25%, binder draindown of less than 0.3%, and abrasion loss that is lower than 15%. Binder contents selected for the samples are from 4% to 7% for abrasion loss and air void samples while, for binder drainage test, the binder content selected is from 5% to 9% at an increment of 1%. Three duplicate samples were produced for each rubber content, percentage, and particle size. The samples are designated according to their rubber mesh size, rubber content, and binder content. For example, 20# 8R 5B represents samples that were modified with 8% crumb rubber sizing 20 mesh at 5% binder content.

### 4.2. Volumetric Properties

In order to calculate the percent of air void in the compacted samples, *P*_av_, volumetric properties of the mixes such as theoretical maximum specific gravity, *G*_mp_, and bulk specific gravity, *G*_bcm_, have to be determined in accordance with D2041 and D3203, respectively. The theoretical maximum specific gravity, *G*_mp_, was calculated from (1), while the bulk specific gravity of each sample was then calculated from (2). Consider

(1) $$\begin{array}{c} G_{\text{mp}}=\frac{A}{A+D-E}, \end{array}$$

where *A* = mass of oven dry sample in air, g, *D* = mass of pycnometer filled with water at 25°C, g, and *E* = mass of pycnometer filled with sample and water at 25°C, g. Consider

(2) $$\begin{array}{c} G_{\text{bcm}}=\frac{A}{B-C}, \end{array}$$

where *A* = mass of the dry specimen in air, g, *B* = mass of the saturated surface-dry specimen in air, g, and *C* = mass of the specimen in water, g.

The air voids (*P*_av_) in a compacted mixture are defined as the ratio between the volume of the small air voids between the coated particles and the total volume of the mixture. It is related to the *G*_bcm_ and *G*_mp_ determined in accordance with the ASTM D2041 and ASTM D3023, respectively. The *P*_av_ value was calculated using the following equation:

(3) $$\begin{array}{c} P_{\text{av}}=100\frac{G_{\text{mp}}-G_{\text{bcm}}}{G_{\text{mp}}}, \end{array}$$

where *G*_mp_ = maximum specific gravity of the compacted mixture and *G*_bcm_ = bulk specific gravity of the compacted paving mixture.

### 4.3. Binder Drainage Test

The binder drainage was conducted based on basket method following a method adapted from BS EN 12697-18:2004. The drained material, *D*, was calculated from

(4) $$\begin{array}{c} D=100\times \frac{\left(W_{2}-W_{1}\right)}{\left(1100+A+B\right)}, \end{array}$$

where *W*_1_ = the initial mass of the tray and foil (gm), *W*_2_ = the mass of the tray and foil with the drained material (gm), *A* = the mass of Portland cement (gm), and *B* = the initial mass of binder in the mixture (gm).

### 4.4. Cantabro Test on Air and Water Cured Samples

The Cantabro Test was performed following a method adapted from D7064. The Cantabro Test was performed to analyze the resistance of compacted porous mixture to abrasion and was carried out in the abrasion machine for 300 revolutions. The percentage of air abrasion loss, *P*, was calculated according to

(5) $$\begin{array}{c} P=\frac{P_{1}-P_{2}}{P_{1}}\times 100, \end{array}$$

where *P*_1_ = initial mass of sample and *P*_2_ = mass of sample after 300 revolutions.

## 5. Results

**Figure 2:**
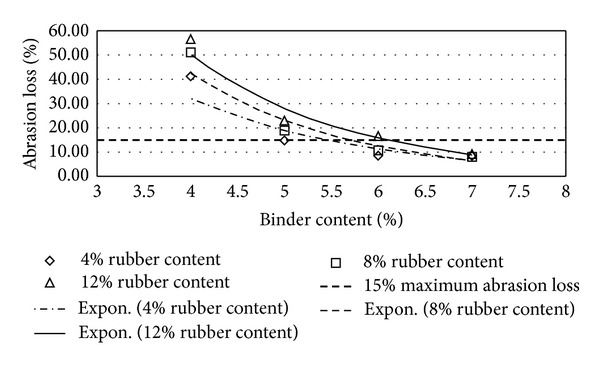
Abrasion loss at different rubber contents for 20 mesh rubber particle size.

**Figure 3:**
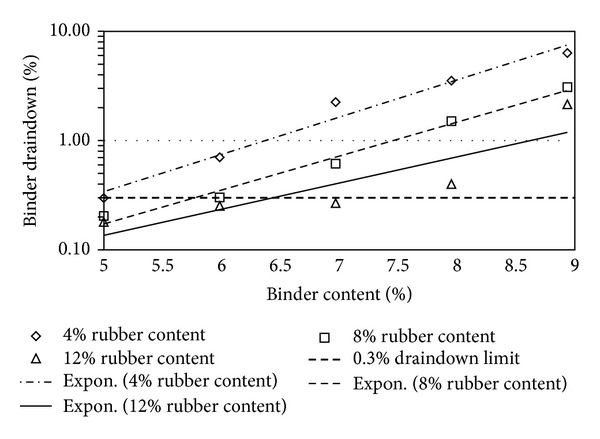
Binder draindown at different rubber contents for 20 mesh rubber particle size.

**Figure 4:**
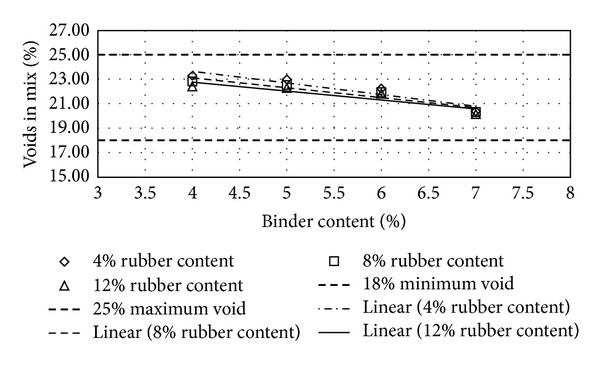
Void in mix (VIM) at different rubber contents for 20 mesh rubber particle size.

**Figure 5:**
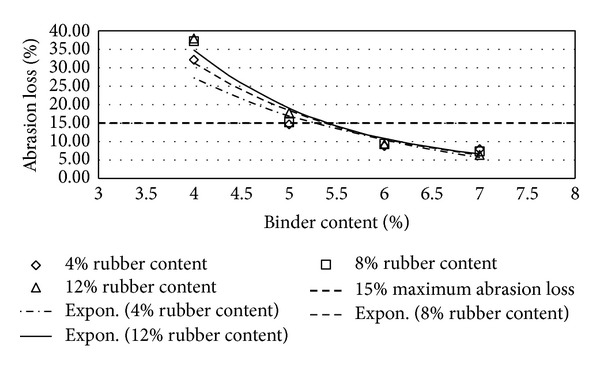
Abrasion loss at different rubber contents for 40 mesh rubber particle size.

**Figure 6:**
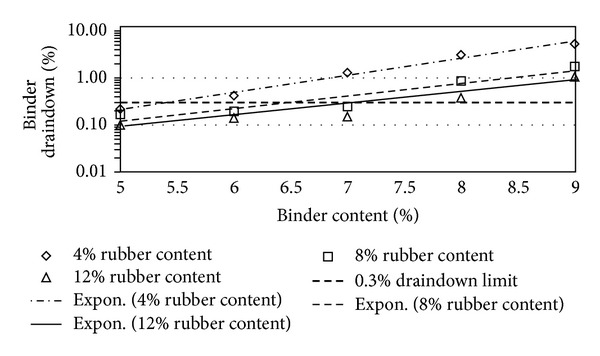
Binder draindown at different rubber contents for 40 mesh rubber particle size.

**Figure 7:**
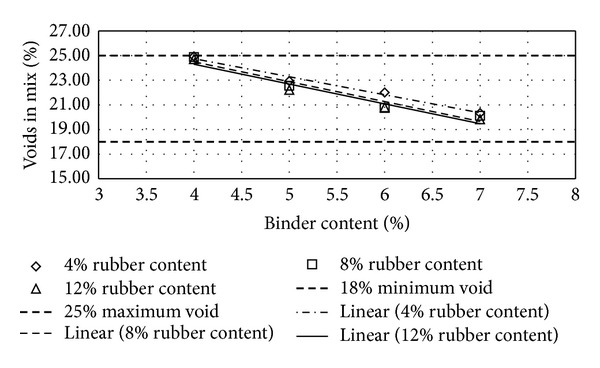
Void in mix (VIM) at different rubber contents for 40 mesh rubber particle size.

**Figure 8:**
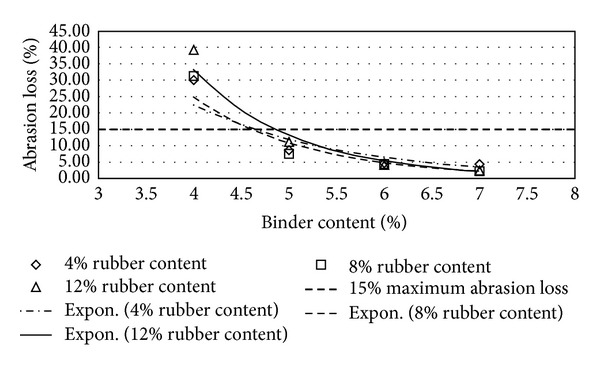
Abrasion loss at different rubber contents for 80 mesh rubber particle size.

**Figure 9:**
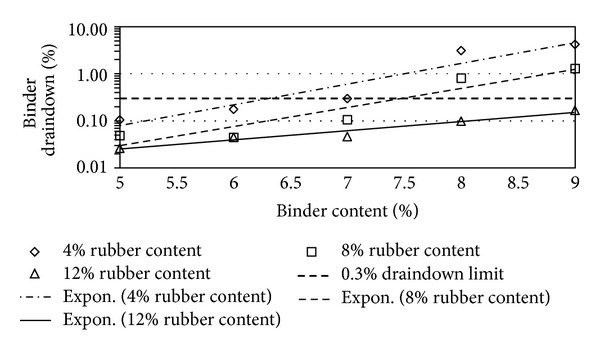
Binder draindown at different rubber contents for 80 mesh rubber particle size.

**Figure 10:**
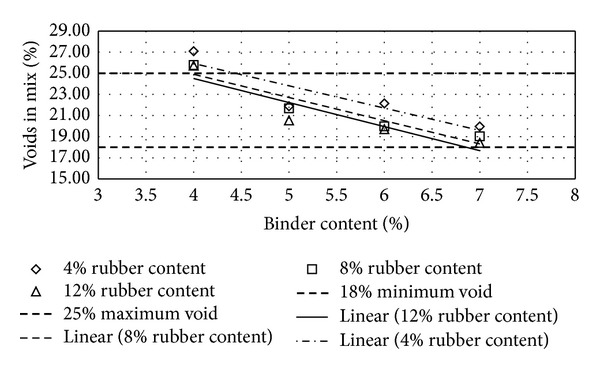
Void in mix (VIM) at different rubber contents for 80 mesh rubber particle size.

**Figure 11:**
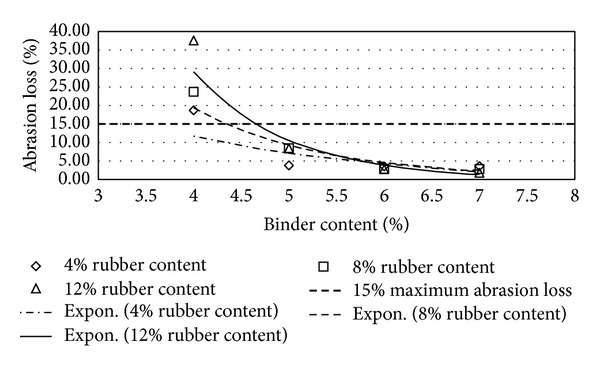
Abrasion loss at different rubber contents for 100 mesh rubber particle size.

**Figure 12:**
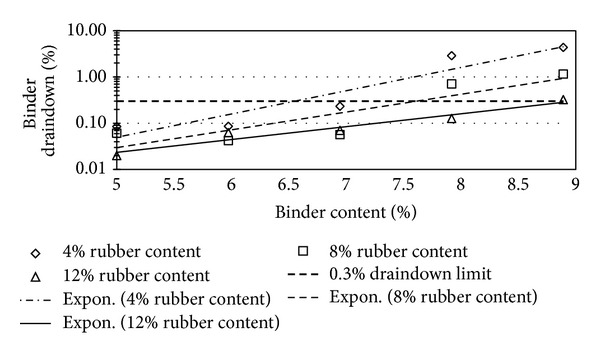
Binder draindown at different rubber contents for 100 mesh rubber particle size.

**Figure 13:**
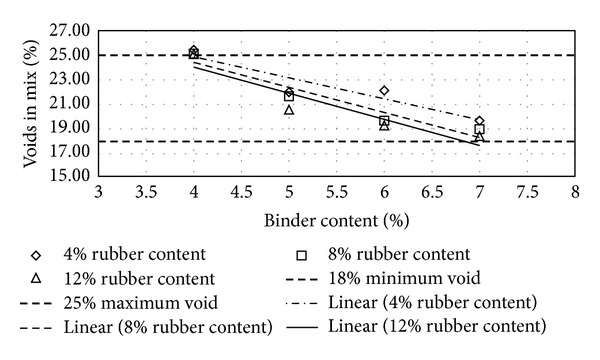
Void in mix (VIM) at different rubber contents for 100 mesh rubber particle size.

Figures 2, 3, 4, 5, 6, 7, 8, 9, 10, 11, 12, and 13 show the abrasion loss, binder draindown, and void in mix pattern for different crumb rubber particle size (RS), rubber content (RC), and bitumen content (BC). The *y*-axis for binder draindown graphs is fitted with a log-scale axis to facilitate the interpretation of the values with exponential pattern. All samples show an exponential trend for abrasion loss and binder draindown results, while air void and binder content seem to indicate a linear relationship.  Table 3 summarizes all the values of the above parameter, highlighting the values that fit the requirement for optimum binder content selection.

### 5.1. Samples with 20 Mesh Rubber Crumb

The abrasion loss for samples prepared with 20 mesh rubber crumb shows an exponential trend for 4% to 7% BC. At 4% BC, abrasion loss is extremely high for all samples reaching 55.6% loss for 12% RC, 51% for 8% RC, and 41.2% for 4% RC. This value drops significantly at 5% BC where the abrasion loss for samples with 12% RC is 23% and, at 5% BC, the average abrasion loss is 14.8%, a permissible value as specified by the REAM Porous Asphalt design guide. Acceptable abrasion loss values are recorded for all samples with 6% and 7% BC with all RC except for samples with 12% RC and 6% BC. This is probably due to inadequate binder in a mix that was modified with a high level of crumb rubber.

Binder draindown tests show very high draindown value for samples prepared with 8% and 9% binder content. At 7% BC, samples with 12% RC show an allowable draindown with a value of 0.27%, while, at 5% and 6% BC, all samples show acceptable value of draindown except for samples modified with 4% RC. Adequate void in mix is acquired for all binder and rubber content.

### 5.2. Samples Modified with 40 Mesh Rubber Crumb

All samples show satisfactory abrasion loss at 6% and 7% binder content for samples modified with 40 mesh rubber crumbs due to the extra strength provided by higher binder content. At 5% BC, only samples with 4% RC show adequate abrasion loss value of less than 15%, while, at 4% BC, none of the samples show acceptable abrasion loss. Again, this is due to a high amount of rubber that is not compensated with higher binder content. This makes the mix brittle and shatters with impact.

Binder draindown at 8% and 9% is too far from the acceptable limit as expected, whereas, at 6% and 7% BC, samples modified with 8% and 12% show draindown value that is within the specified limit. At 5% BC, all samples gave acceptable draindown values. Contrary to the factors that govern abrasion loss, binder draindown value will increase as lower RC is used with samples that have a higher BC. This results in binders that have a low viscosity and tend to draindown easily. Void in mix still shows satisfactory value for all binder and rubber content tested.

### 5.3. Samples Modified with 80 Mesh Rubber Crumb

Due to the finer nature of the rubber crumb, 80 mesh crumb rubber modification provides an acceptable abrasion loss for samples at all binder content except at 4% BC. Finer rubber crumb has larger surface area and hence absorbs higher amount of lighter oils in the bitumen. This increases the viscosity of the binder and subsequently increases the strength of the mix.

Draindown characteristics, on the other hand, give sufficient results for 5%, 6%, and 7% BC. At 8% and 9% BC, only samples with 12% RC provide adequate draindown value. As with coarser mesh size, this can be expected as 8% and 9% BC lower RC cannot provide the higher viscosity that is required to give acceptable draindown value.

Void in mix results show a slightly different pattern compared to previous results. At 4% binder content, the amount of void in the mix does not satisfy the requirement of the specification. This shows that finer rubber crumb would require a higher bitumen content to give the bitumen a viscosity that is low enough to be able to fully coat the aggregate but at the same time the viscosity is not too low that it drains down easily. Lower binder content combined with rubber crumb modification would produce a binder that is too thick and would not provide good coverage to the aggregates, hence creating higher void in the mix.

### 5.4. Samples Modified with 100 Mesh Rubber Crumb

Samples modified with 100 mesh crumb rubber are showing similar results with its 80 mesh counterpart. This is probably because the difference in size between the two mesh sizes is probably not significant.

### 5.5. Selection of Optimum Binder Content

Table 3 summarizes the average value for the abrasion loss, binder draindown, and void in mix for all the rubber size, content, and binder content considered in this study. Values that satisfy all the criteria required to select the optimum binder content were highlighted in bold. From the table, it is observed that samples that were modified with 20 mesh rubber display narrower window for optimum binder content selection. At 4% rubber content, 5% BC was found to provide adequate abrasion loss, binder draindown, and void in mix, while, for 8% and 12% RC, 6% BC, and 7% BC, was found to satisfy the specification requirement, respectively. Mixes with 40 mesh rubber have a slightly wider selection of optimum binder content. At 8% and 12% RC, optimum binder for the samples can be selected between 6% and 7%. However, at 4% RC only 5% BC can be selected as the optimum binder content.

Binder contents of 5%, 6%, and 7% can be chosen for optimum binder content for mixes modified with finer rubber crumb sizing 80 and 100 mesh size. This is interesting as one would expect that finer rubber crumbs and higher binder content would produce a stiffer mix with a lower void content. Although the void content does decrease and the draindown value increases, the value of the parameters are still within acceptable standards for a wider binder content range.

## 6. Conclusion

Analyzing the results of the tests, the following conclusions can be drawn.

1. Mixes modified with coarser rubber crumb and lower bitumen content tend to have a higher abrasion loss. This is due to inadequate binder content for compensate to the addition of rubber crumb resulting in a mix that is brittle and shatters upon impact.
2. Coarser rubber crumb has a narrower window of selection of optimum binder content. As the mesh size decreases, higher binder and crumb rubber content can be used. However, it is important to note that this study uses low shear mixing of rubber crumbs at reduced mixing temperature; therefore, it can be anticipated that higher shear mixing and higher mixing temperature can change this nature of coarser rubber crumbs as higher blending temperature and high shear mixing tend to increase the swelling and reduce the size of the crumb rubber.
3. While extra bitumen provides higher strength, mixes with higher binder content are subject to greater binder draindown value. Higher rubber content also increases the viscosity of bitumen which also increases the film thickness subsequently lowering the void in mix. However, finer rubber crumbs seem to be less sensitive to the increment of rubber and binder content. This results in a wider window for selection of the optimum binder content.
4. The flexibility in the range of optimum binder content provided by bitumen modified with finer rubber crumb (80 and 100 mesh size) allows a wider range of optimum binder content selection. However, the lower and the upper limit of the optimum binder content (5% and 7%) lies in close proximity to the maximum and minimum limit of the allowable void content of 18% and 25%. Therefore, for practical use, an intermediate value between 6% and 6.5% is advisable to be used as the optimum binder content to allow a certain level of tolerance when producing rubberized bitumen and at the same time ensuring a high quality product.

## Figures and Tables

**Table 1:** Parameter used in determining optimum binder content for open graded friction courses [2, 7].

Authority	Air voids (%)	Abrasion loss (%)	Binder draindown (%)
ASTM (USA)	≥18	≤20	≤0.3
NCAT (USA)	≥18	≤20	≤0.3
Virginia Department of Transport (USA)	≥16	≤20	≤0.3
REAM (Malaysia)	18%–25%	≤15	≤0.3
Austroads (Australia)	20%–25%	≤20	≤0.3

**Table 2:** Grading type B as specified in Malaysian Guide for Porous Asphalt REAM-SP 5/2008.

Sieve size (mm)	Percent passing
20.00	100.0
14.00	85–100
10.00	55–75
5.00	10–25
2.36	5–10
0.075	2–4

**Table 3:** Summary for the values of abrasion loss, binder draindown, and void in mix.

Sample	Average abrasion loss (%)	Average binder draindown (%)	Average void in mix (%)	Sample	Average abrasion loss (%)	Average binder draindown (%)	Average void in mix (%)
20# 4R 4B	41.19	—	23.28	80# 4R 4B	29.97	—	27.08
20# 4R 5B	**14.81**	**0.30**	**22.97**	80# 4R 5B	**8.61**	**0.10**	**21.87**
20# 4R 6B	**8.51**	0.70	**22.22**	80# 4R 6B	**4.71**	**0.18**	**22.15**
20# 4R 7B	**8.47**	2.25	**20.36**	80# 4R 7B	**4.40**	**0.30**	**19.95**
20# 4R 8B	—	3.53	—	80# 4R 8B	—	3.10	—
20# 4R 9B	—	6.32	—	80# 4R 9B	—	4.19	—
20# 8R 4B	51.04	—	22.81	80# 8R 4B	31.22	—	25.77
20# 8R 5B	18.89	**0.20**	22.52	80# 8R 5B	**7.55**	**0.05**	**21.67**
20# 8R 6B	**10.68**	**0.30**	**21.93**	80# 8R 6B	**4.41**	**0.04**	**19.97**
20# 8R 7B	**8.06**	0.61	**20.30**	80# 8R 7B	**2.22**	**0.11**	**19.03**
20# 8R 8B	—	1.50	—	80# 8R 8B	—	0.80	—
20# 8R 9B	—	3.09	—	80# 8R 9B	—	1.28	—
20# 12R 4B	56.56	—	22.41	80# 12R 4B	39.14	—	25.73
20# 12R 5B	23.00	**0.18**	22.27	80# 12R 5B	**11.15**	**0.03**	**20.54**
20# 12R 6B	16.60	**0.25**	21.81	80# 12R 6B	**4.16**	**0.04**	**19.70**
20# 12R 7B	**9.16**	**0.27**	**20.13**	80# 12R 7B	**2.43**	**0.05**	**18.41**
20# 12R 8B	—	0.40	—	80# 12R 8B	—	0.10	—
20# 12R 9B	—	2.15	—	80# 12R 9B	—	0.17	—
40# 4R 4B	32.14	—	24.93	100# 4R 4B	18.67	—	25.43
40# 4R 5B	**14.66**	**0.22**	**22.92**	100# 4R 5B	**3.80**	**0.09**	**21.99**
40# 4R 6B	**8.76**	0.42	**22.00**	100# 4R 6B	**3.57**	**0.09**	**22.13**
40# 4R 7B	**7.96**	1.29	**20.34**	100# 4R 7B	**3.58**	**0.23**	**19.68**
40# 4R 8B	—	3.11	—	100# 4R 8B	—	2.88	—
40# 4R 9B	—	5.29	—	100# 4R 9B	—	4.36	—
40# 8R 4B	37.18	—	24.87	100# 8R 4B	23.65	—	25.12
40# 8R 5B	15.20	**0.17**	22.55	100# 8R 5B	**8.44**	**0.06**	**21.67**
40# 8R 6B	**9.42**	**0.20**	**20.75**	100# 8R 6B	**2.88**	**0.04**	**19.68**
40# 8R 7B	**7.33**	**0.25**	**20.11**	100# 8R 7B	**2.84**	**0.06**	**18.99**
40# 8R 8B	—	0.86	—	100# 8R 8B	—	0.71	—
40# 8R 9B	—	1.74	—	100# 8R 9B	—	1.15	—
40# 12R 4B	37.88	—	24.71	100# 12R 4B	37.51	—	25.09
40# 12R 5B	17.78	**0.10**	22.21	100# 12R 5B	**8.36**	**0.02**	**20.57**
40# 12R 6B	**9.31**	**0.14**	**20.80**	100# 12R 6B	**2.79**	**0.06**	**19.30**
40# 12R 7B	**6.37**	**0.15**	**19.82**	100# 12R 7B	**1.82**	**0.07**	**18.43**
40# 12R 8B	—	0.38	—	100# 12R 8B	—	**0.13**	—
40# 12R 9B	—	1.05	—	100# 12R 9B	—	0.32	—
